# Bone gain and accuracy assessment of computer-guided workflow for horizontal augmentation of atrophic anterior maxilla with symphyseal cortical plates: a randomized controlled trial

**DOI:** 10.1186/s12903-025-06415-2

**Published:** 2025-07-02

**Authors:** Dina Ayman, Mohamed Shawky, Lobna Abdel Aziz Aly, Mohamed Mounir, Ahmed K. Abo Zekry

**Affiliations:** 1 Department of Oral and Maxillofacial Surgery, Faculty of Oral and Dental Medicine, Future University in Egypt, Cairo, Egypt; 2 Department of Oral and Maxillofacial Surgery, Faculty of Dentistry, Cairo University, PO Box 11553, Cairo, Egypt; 3 Division of Oral and Maxillofacial Surgery, School of Dentistry, New Giza University, Giza, Egypt

**Keywords:** Bone transplantation, Bone shell, Bone plates, Alveolar bone loss, Computer-aided design, Maxilla, Mandible, RCT

## Abstract

**Background:**

Horizontal augmentation of the anterior maxilla is a highly demanding and yet predictable procedure. This study aimed to investigate the efficiency and accuracy of a full computer-guided symphyseal shell harvesting and positioning approach versus the conventional procedure for the treatment of horizontally atrophic anterior maxillary ridges.

**Patients and methods:**

Twenty patients with horizontally deficient anterior maxilla were randomly allocated into two groups, ten patients each. The study group received a fully guided horizontal augmentation of their atrophic anterior maxillary ridges using symphyseal shells. While the control group received the same treatment but without surgical guides. Bone gain was calculated for both groups and the accuracy of the computer guidance was investigated in the study group.

**Results:**

After four months of graft consolidation, there was no statistically significant difference between both groups regarding the horizontal bone gain and the mean bone gain percent, measuring a mean of 3.66 ± 0.63 mm and 105.71% ± 29.75 mm in the guided group while for the control group, it measured 3.33 ± 1.07 mm and 90.41% ±31.77 mm respectively (P-values = 0.4 and 0.28). In the study group, there was no statistically significant difference between the planned and the achieved horizontal augmentation (*p* = 0.97).

**Conclusion:**

Despite the lack of statistically significant difference between both groups with regard to horizontal bone gain, computer-guided cortical shell technique allowed for accurate, simple, and safe graft positioning and fixation; it is worth further investigations to explore its different applications.

**Trial registration:**

Retrospectively registered on www.clinicaltrials.gov with ID: NCT05311332 on 27-03-2022.

**Clinical trials.gov registered ID:**

NCT05311332.

**Supplementary Information:**

The online version contains supplementary material available at 10.1186/s12903-025-06415-2.

## Introduction

Implant-supported restorations have become the standard means of rehabilitation of missing teeth [1]. Regardless of the many reasons for teeth loss, which range from destructive decay to periodontal disease or dentoalveolar trauma and pathological lesions, the masticatory function is negatively affected and in the anterior maxillary region, esthetics is compromised. Moreover, the mechanical stimulation of the alveolar process is lost leading to disuse atrophy and making implant placement more challenging later [2].

For prosthetically favorable implant placement and better esthetic outcomes, dental implants should be surrounded by 1.5 to 2 mm of bone on the buccal and palatal sides [3]. If a 4 mm-diameter implant is used, this means that 7 to 8 mm of bone width is needed [4]. To augment horizontally atrophic anterior maxillary alveolar ridges to the desired bone parameters, many surgical procedures and techniques could be used, such as onlay grafting, ridge splitting, and guided bone regeneration using resorbable or non-resorbable barriers [5–7].

It is widely accepted that autogenous bone grafts are the gold standard due to their osteogenic, osteoconductive, and osteoinductive capabilities. Several donor sites were reported in the literature, extraoral sites are sought when large augmentations are unavoidable and they include the iliac crest, parietal bones and tibia [8]. Otherwise the intraoral donor sites such as the symphyseal region and the ascending ramus are more widely explored due to their proximity to the recipient sites and much less morbidity compared to the extraoral sites [9]. Regarding the embryologic origin, intramembranous donor sites– which include the chin and ascending ramus - are characterized by better volumetric stability in bone augmentation procedures [10, 11, 12, 13].

The chin region is characterized by being abundant in bone reserve when compared to the retromolar region, symphyseal bone blocks are harvested with a thick spongy layer that adds to the overall thickness of the graft and is advantageous in onlay grafting procedures [14]. Moreover, larger volumes of bone could be harvested from the chin which makes is favorable when relatively large grafts are needed [15]. Regarding anterior maxillary recipient sites, the natural curvature of the mandibular symphysis is ideal as it follows the contour of the anterior maxilla [16].

In contrast to the onlay technique described by Michael Pikos [17]the thin cortical shells harvested from the retromolar area described by Fouad Khoury offered less time for revascularization and remodeling. Acting as a natural barrier and maintaining a space between the native bone and the fixed cortical shell to be obliterated by particulate bone graft [1]. The autogenous cortical shell technique is predictable, and it could provide an appropriate bone volume and architecture, which are key requirements for successful dental implant insertion in a desirable prosthetic position [16, 18].

Despite its many advantages, it is technique-sensitive, requiring a steep learning curve as it depends on the operator’s experience during harvesting and fixation to avoid injury to adjacent vital structures and nerves and to properly place and fix the cortical shell [18]. Additionally, the anterior maxillary ridge is quite unique; there is no anatomical guidance during shell fixation. The only way to determine the desired gap between the ridge and the shell is through the experience of the surgeon, and hence some mishaps could occur, such as deficient augmentation or over-enhancement of the ridge and sometimes malpositioned or rotated fixation [19].

With the advent of CAD/CAM technology, several procedures were facilitated and performed with great accuracy and reduced intraoperative time such as computer-guided symphyseal bone harvesting [7]ridge splitting using patient-specific guides [20]patient-specific titanium meshes or zirconia shells for guided bone regeneration to decrease the duration of the operation and enhance the regenerated bone volume [21, 22]and the computer-guided sandwich osteotomy technique for vertical ridge augmentation [23].

The recent initiative by Tonetti et al. to develop core outcome set and measurements for implant dentistry clinical trials (ID-COSM) identified specific mandatory outcomes for bone augmentation studies [24]. Identified as bone augmentation core outcome set and measurements (BA-COSM) they included; surgical complications, bone dimensional changes and the ability to place the dental implant in a favorable position for subsequent prosthetic loading. Following this initiative, and since that the symphyseal region follows the natural curve and contour of the anterior maxillary ridge to a great extent, the present study aimed to investigate the amount of bone gain and accuracy of a fully computer-guided horizontal augmentation of atrophic anterior maxillary ridges using cortical shells harvested from the mandibular symphysis along with assessment of the effect of the surgical approach on the neurosensory recovery of the mental nerves.

## Patients and methods

### Trial design

This randomized clinical trial was approved by the research ethics committee, faculty of oral and dental medicine, Future University in Egypt, with the reference number: (23)11-2021. It was registered on clinicaltrials.gov in April 2022 (https://clinicaltrials.gov/study/NCT05311332) with the ID: NCT05311332, followed the Declaration of Helsinki regarding the ethical principles for medical research involving human subjects and was written according to the CONSORT guidelines. Enrollment commenced in April 2022.

Sample size calculation was performed using G Power statistical tool (version 3.1.9.4) according to a previous study on maxillary horizontal alveolar ridge augmentation by [20]. Based on an effect size (ES) of 1.68 and assuming an 80% power, with a significant level (α error) of 0.05 for a two-tailed hypothesis. A total sample size of (*n* = 14, subdivided into 7 in each study group) was calculated. The number of participants recruited for each group was raised to 10 in each group (i.e. total sample size *n* = 20) to maintain statistical power in case of any dropout cases that may have occurred during the course of the trial period.

After being assigned coded numbers that were concealed in opaque, sealed envelopes, the patients were randomly allocated into two equal groups using block randomization with stratification (block:4) using a formula on Microsoft Excel software by an independent investigator who was not one of the authors. The operator and the patient were blinded until they were informed about the allocation of each case just before the operation started. However, the outcome assessor and the statistician who analyzed the collected data and performed the statistical analysis were blinded. All operations were performed by the same surgeon (DA) in both groups in the first stage and the second stage interventions. In the study group, horizontal augmentation of atrophic anterior maxillary ridges in ten patients was done with an entirely computer-guided approach using two bone-supported surgical guides: the first one for harvesting exactly the needed dimensions of the symphyseal cortical shell while protecting the adjacent vital structures, and the second one for fixing the shell in its exact pre-planned position. In the control group, the symphyseal cortical shells were harvested and fixed in a conventional way without any surgical guides.

### Recruitment

From both Cairo University’s and the Future University’s outpatient clinics of the departments of oral and maxillofacial surgery, patients with the following inclusion criteria were recruited: Adult patients of both genders with ages ranging from 20 to 50 years old, suffering from atrophic anterior maxillary alveolar ridges with a bone width less than 3 mm and a minimum bone height of 10 mm and good oral hygiene. While diabetic patients, those with bone metabolic disorders such as osteoporosis, vitamin D deficiency and with a previous history of failed augmentation procedures were excluded. Patients following the inclusion criteria were interviewed to obtain detailed medical and dental histories, clinically examined, and an orthopantomogram was requested from them to exclude any undetected pathologies. The treatment plan was explained to those who agreed to participate, and informed consents were signed.

### Virtual planning and guide fabrication

A cone beam computed tomographic scan (CBCT) (Planmeca, ProMax 3D MAX, Helsinki, Finland) was carried out for each patient to measure the preoperative ridge width and confirm the patient’s eligibility for the study. In the computer-guided group, it was used for virtual planning, where the DICOM files (Digital Imaging and Communication in Medicine) were imported to Mimics 21.0 software (Materialise, Leuven, Belgium). Segmentation was done to isolate the maxilla and create a 3D model, then export it to 3-matic 13.0 software (Materialise, Leuven, Belgium), where the surgical stents were designed.

**Fig. 1:**
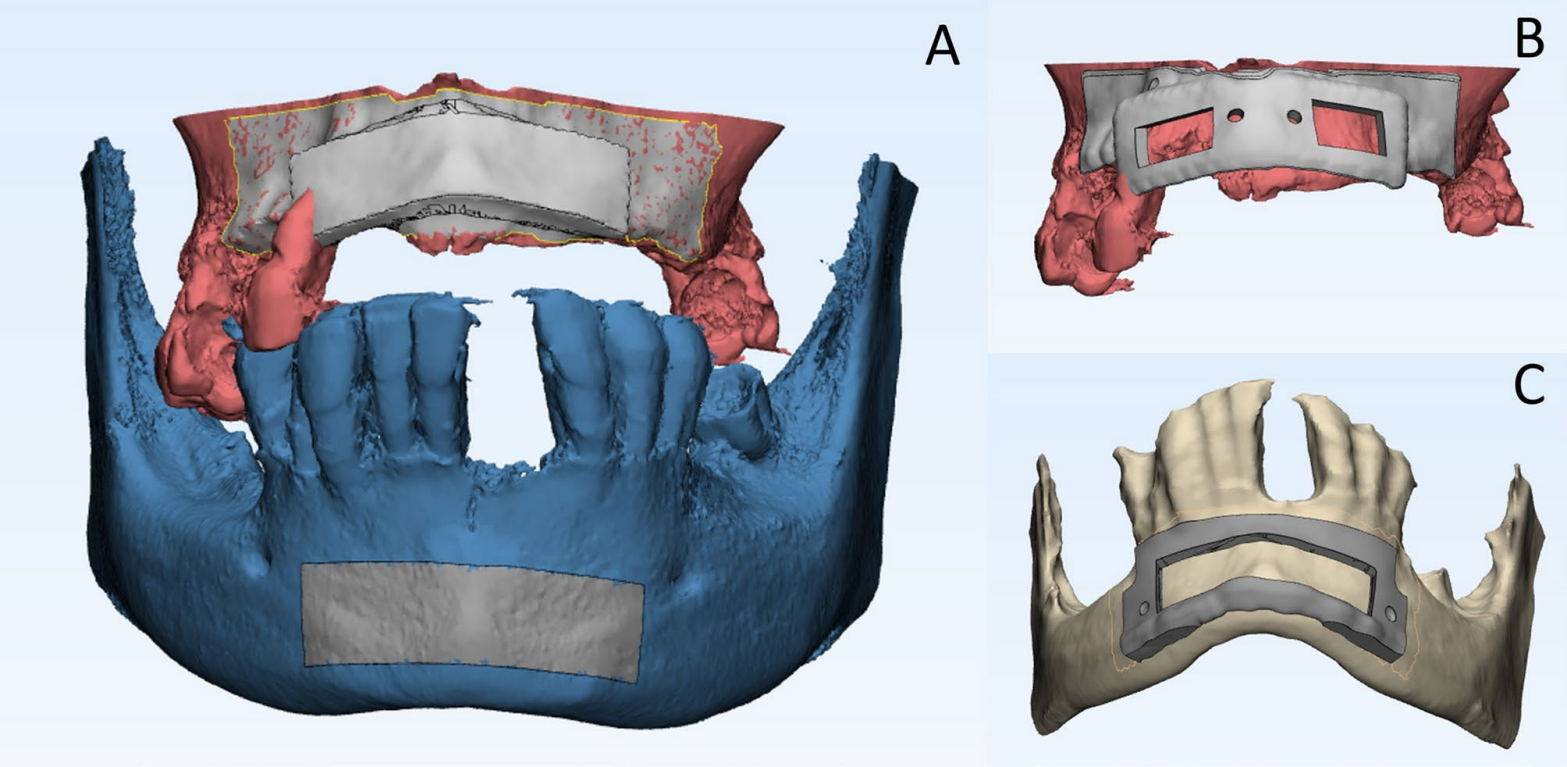
Preoperative planning for the study group **A**) The estimated virtual dimension and position of the bone shell. **B**) Showing the positioning guide at the recipient site, notice the two open windows for block fixation **C**) Harvesting Guide at the donor site

The first surgical guide aimed to guide the harvest of the chin autogenous cortical shell with predetermined dimensions according to the recipient site in each case, taking care to locate the osteotomies at least 5 mm away from the root apices, the inferior border, and the mental foramina bilaterally. The second surgical guide was designed to guide the fixation of the bone shell by positioning it at a predetermined distance from the ridge, where a total of 7–8 mm bucco-palatal width was achieved, and at the same time allow for a through-and-through fixation of the harvested shell to the deficient ridge in its final 3D position via two open windows. (Fig. 1)

Finally, the guides were exported in STL (standard tessellation language) format to be 3D printed from resin using additive manufacturing technology (Form 3B, Formlabs Inc., Somerville, MA). They were sterilized by placing them in a 2% glutaraldehyde solution (Cidex, Johnson & Johnson Co., NJ, USA) for 12 h before surgery, followed by rinsing with normal saline.

### Surgical procedure

Patients were instructed to rinse with 0.2% chlorhexidine mouthwash (Orovex, Macro Group Pharmaceuticals, Cairo, Egypt). The recipient site was approached first, so infraorbital and nasopalatine nerve block techniques were used for pain control in addition to circumferential infiltration for hemostasis. A full-thickness three-line mucoperiosteal flap extending at least two teeth on each side of the area of interest was raised, with a para-crestal incision to the palatal side and two oblique releasing incisions. The buccal cortical plate was fenestrated with a small round bur to enhance vascularity, then periosteal release and flap advancement were done for tension-free closure.

Approaching the donor site started with bilateral mental nerve blocks as well as circumferential infiltration, then a vestibular approach extending between the canines and 5 mm away from the mucogingival junction that was opened in two planes. The chin was exposed down to the inferior border, then for the study group, the harvesting guide was placed and fixed as planned using two monocortical 2.0 mm mini-screws. Diamond disks (Komet Dental, Lemgo, Germany) were used to cut the outline of the cortico-cancellous block following the guide window. The osteotomies were executed in a converging angulation to facilitate both the separation of the graft and its thinning later on. The guide was removed, and the shell separated by gentle malleting, then it was thinned by grinding from its inner side with a large diamond-round stone. The same was done for the control group, but without using a harvest guide.

Then, for both groups, an auto-chip maker bur (ACM, Neo Biotech, South Korea) was used to harvest cortical particulate bone that was mixed in a 1:1 ratio with xenograft (OneGraft, Berlin, Germany). The donor site was packed with a surgical sponge (SURGISPON, Aegis Lifesciences, Gujarat, India) and sutured in layers using a 3 − 0 polyglycolic acid suture (Assucryl, Assut, Switzerland).

**Fig. 2:**
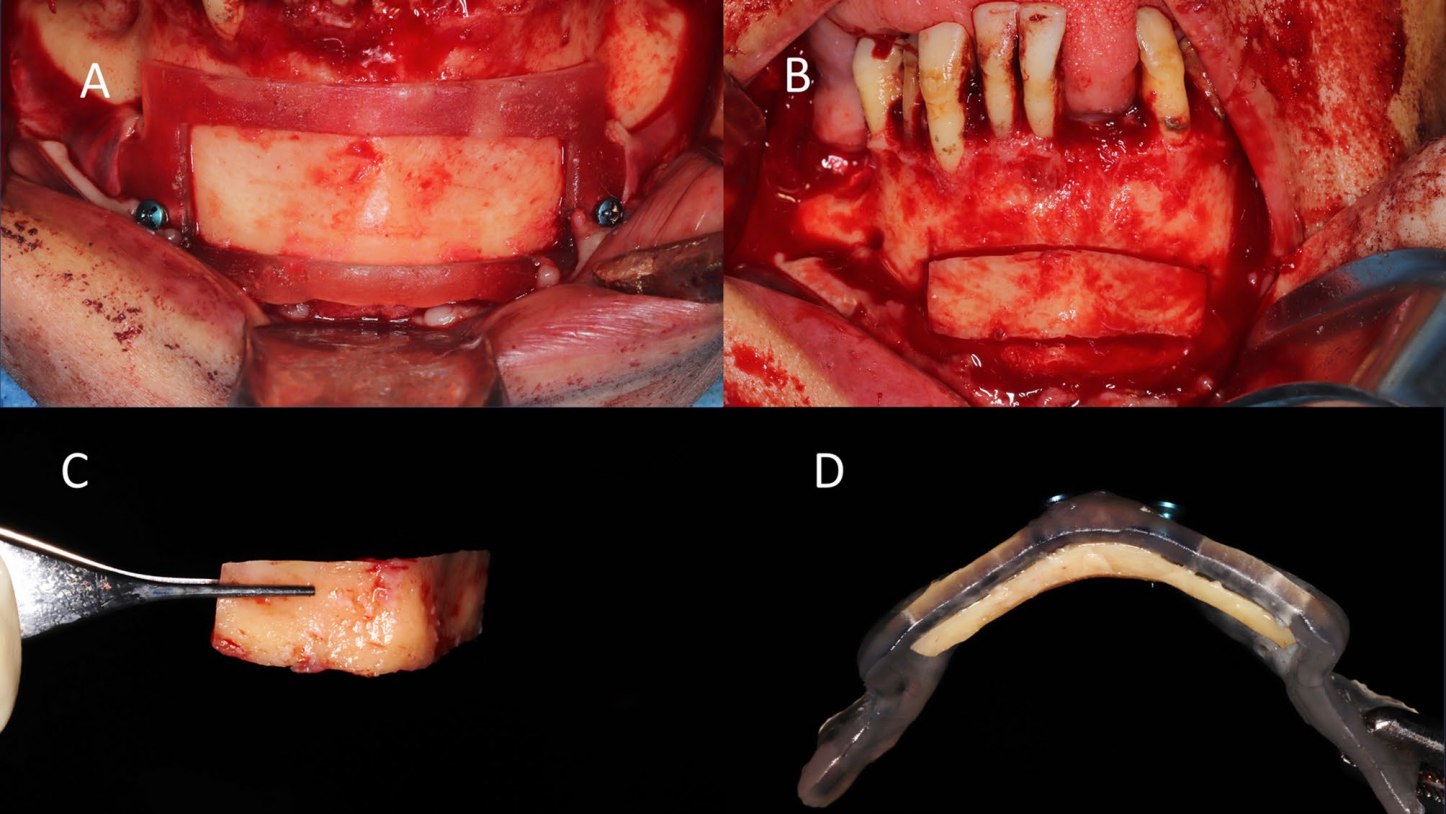
Intra operative procedures for the study group **A**) Donor site with the harvesting guide fixed in place. **B**) Delineation of the shell outline. **C**) Harvested bone shell, **D**) intimate Cortical shell fixation to the second positioning guide using 2 mono-cortical screws

**Fig. 3:**
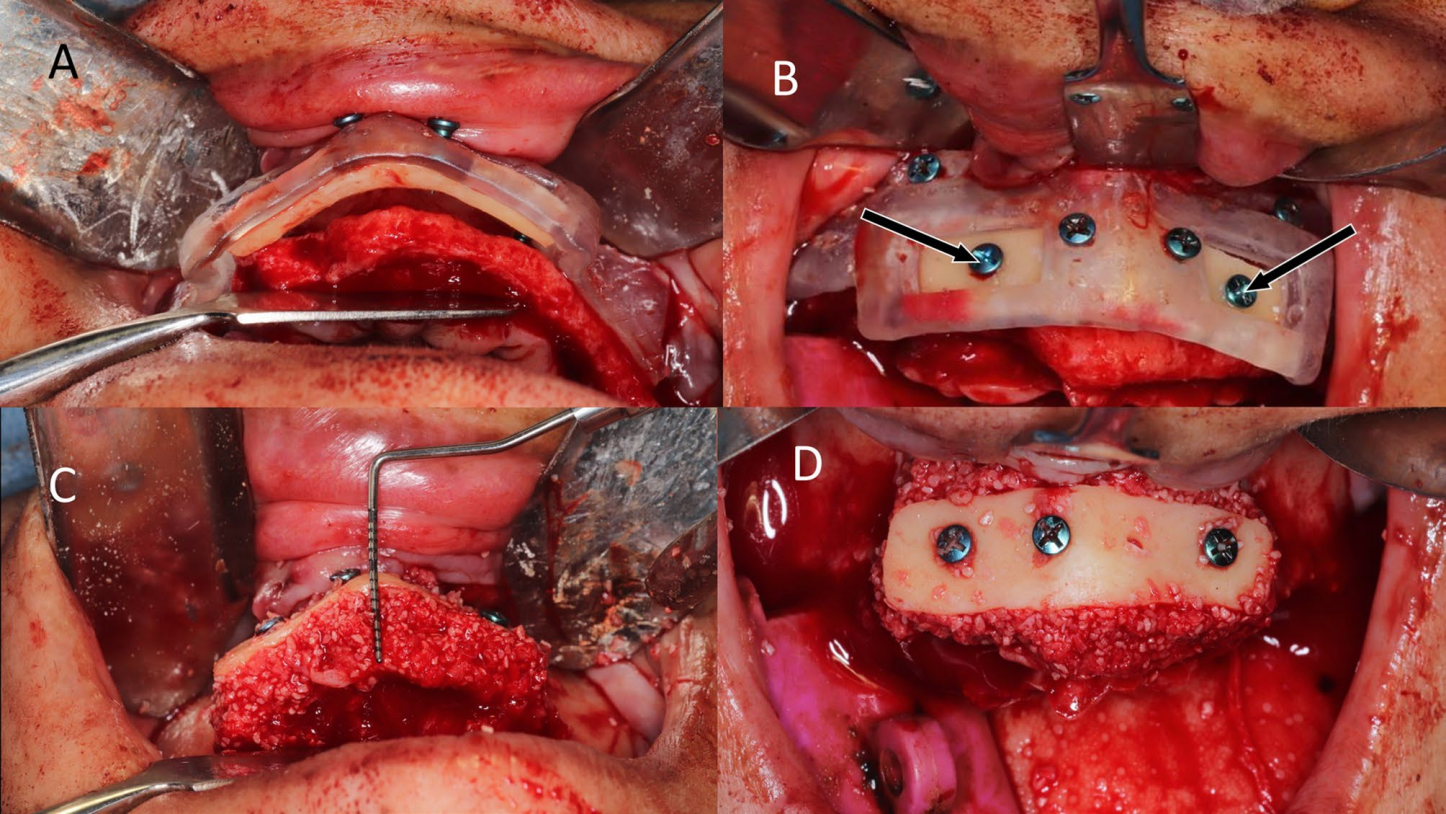
Intra operative procedures for the study group **A**) positioning guide fixed in place at the recipient site and locating the bone shell in its preplanned position, ready for fixation, **B**) Black arrows marking the 2 Bi-cortical screws used for fixation of the shell in a through and through manner to the deficient ridge via the preplanned opened windows. **C**) and **D**) are occlusal and labial views showing the interpositional gap obliterated with particulate bone graft

**Fig. 4:**
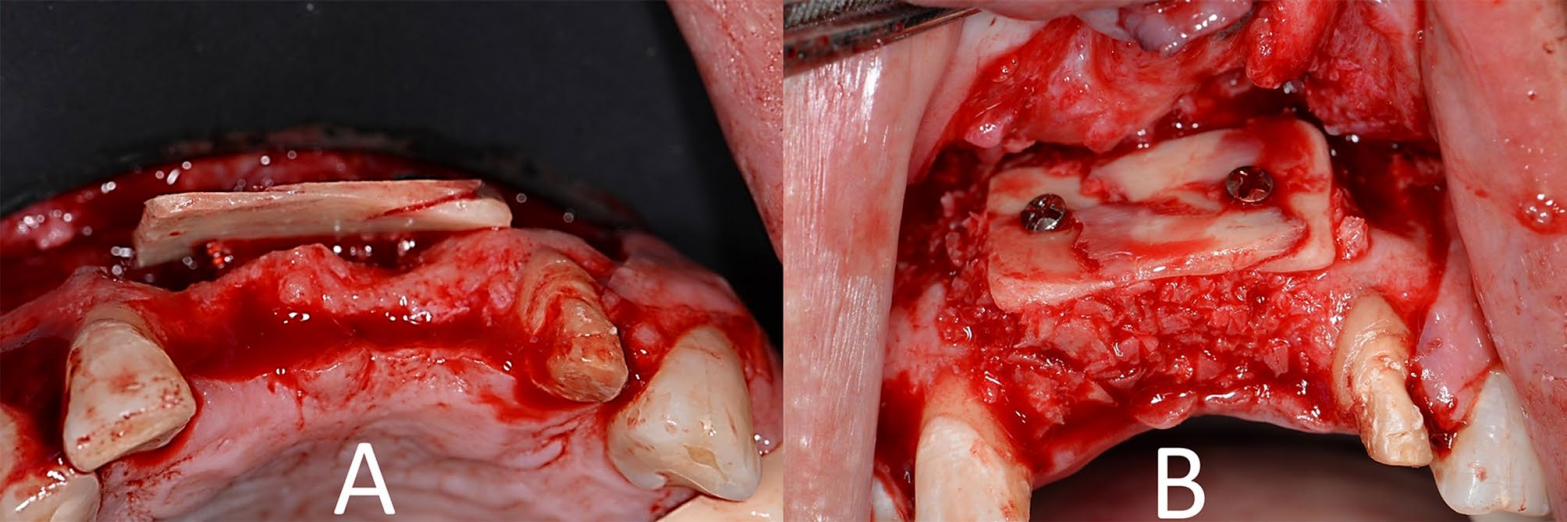
Intra operative procedures for the control group. **A**) Free Hand Harvesting And fixation of the cortical shell leaving the desired gap at the recipient site **B**) particulate bone graft filling the interpositional gap (occlusal view)

In the study group, the harvested cortical shell was fixed to the fitting surface of the positioning guide first outside the patient’s mouth with two micro screws (Fig. 2). Then the positioning guide carrying the cortical shell was installed in its pre-planned position at the recipient site and fixed to the surrounding bone in the pre-designed screw vents. The now-positioned cortical shell in its desired pre-planned position was fixed through the two open windows using two micro-screws, and then the guide was removed. Rounding of any sharp edges was done under copious irrigation (Fig. 3), (Video 1) and the interpositional gap was filled with the particulate mixture. The same was done in the control group, but without using positioning guides. (Fig. 4).

In both groups, suturing was done using a 4 − 0 monofilament, non-resorbable material (Polyproline, Assut, Switzerland) in two planes: apical horizontal mattresses and simple interrupted at the flap margins. An immediate postoperative CBCT scan was ordered from the study group patients for assessment for accuracy.

### Second stage surgery

**Fig. 5:**
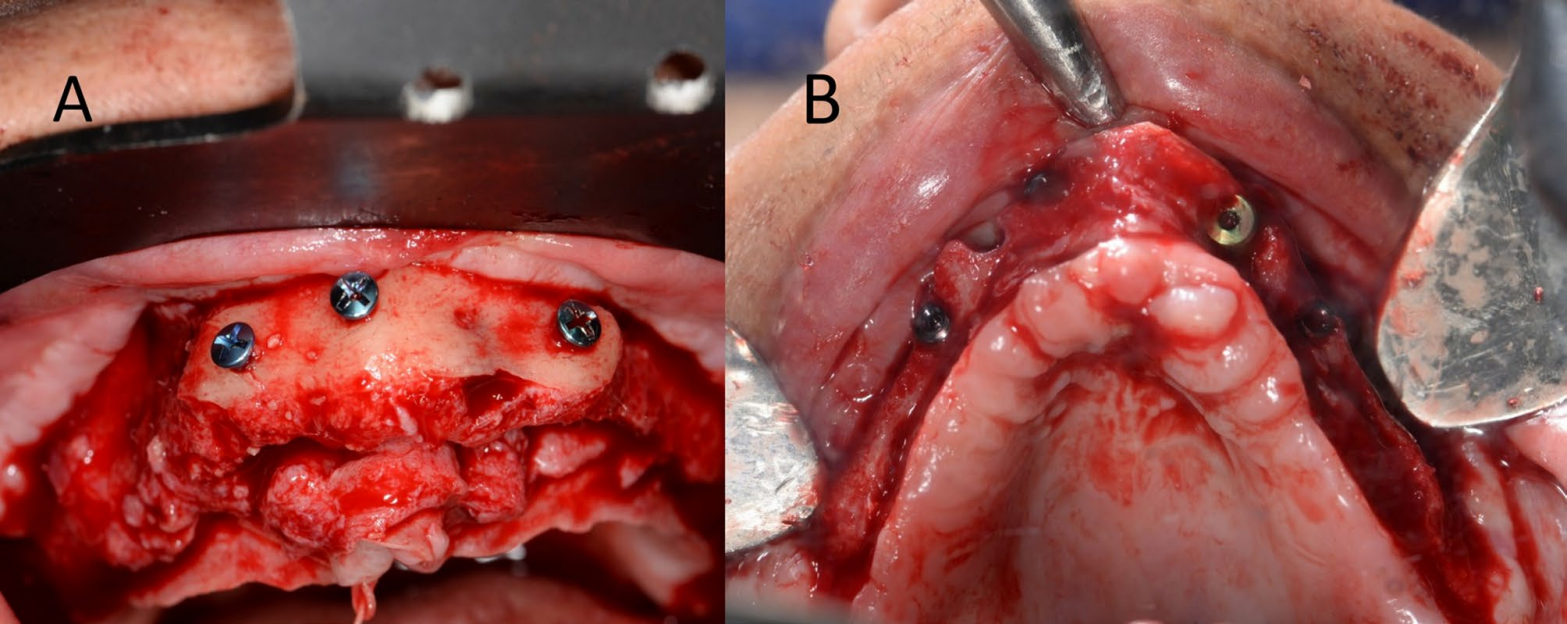
**A**) Second stage surgery in the study group: exposure before removal of fixation screws. **B**) implant placement (occlusal view)

**Fig. 6:**
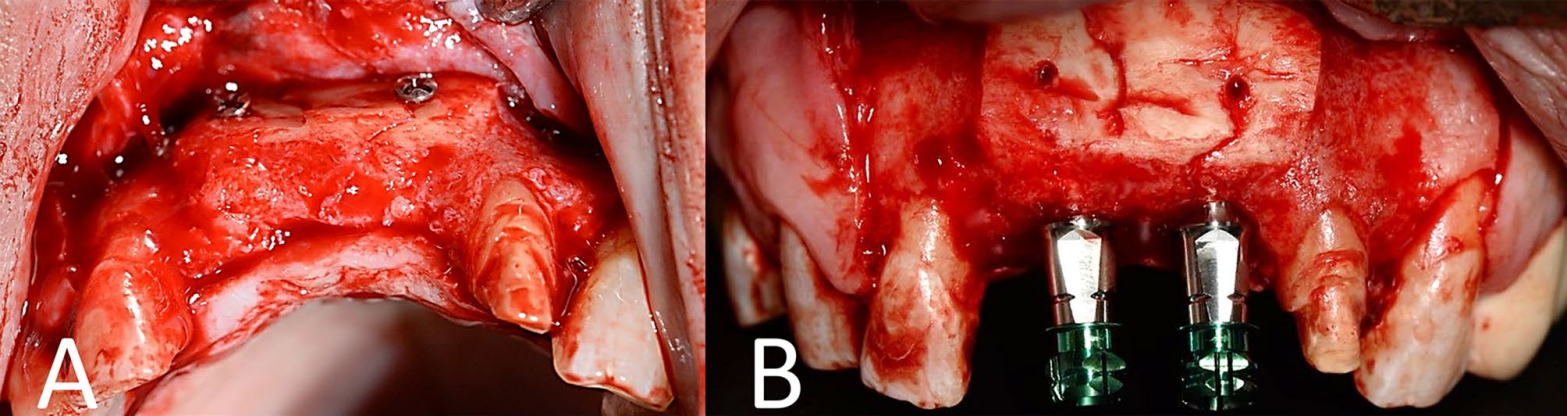
**A**) Second stage surgery in the control group exposure before removal of fixation screws. **B**) implant placement (labial view)

After four months of graft consolidation, CBCT scans were ordered in both groups to determine the new ridge dimensions for implant placement. Following the removal of the fixation screws, a total of 45 dental implants (Dual Implants, Titan Industries, Cairo, Egypt) were successfully placed in a favorable position for prosthetic loading in all cases while preserving a sufficient thickness of buccal and palatal bone, minimum of 1–1.5 mm (Figs. 5 and 6).

To *radiographically* assess the bone width changes around the dental implants, CBCT scans were performed after implants insertion to act as a base line that was compared to new CBCT scans that were ordered at the time of implants exposure for prosthetic loading (four months after implantation). All implants were found to be successfully integrated and the final prosthetic loading was done using FP1 fixed zirconia restorations for all patients in both groups. Moreover, after one year (one year after prosthetic loading), the pink esthetic score (PES) *clinically assessed* the gingival and biological integrity around each implant in both groups, the mean for each case was calculated.

### Postoperative assessment

**Fig. 7:**
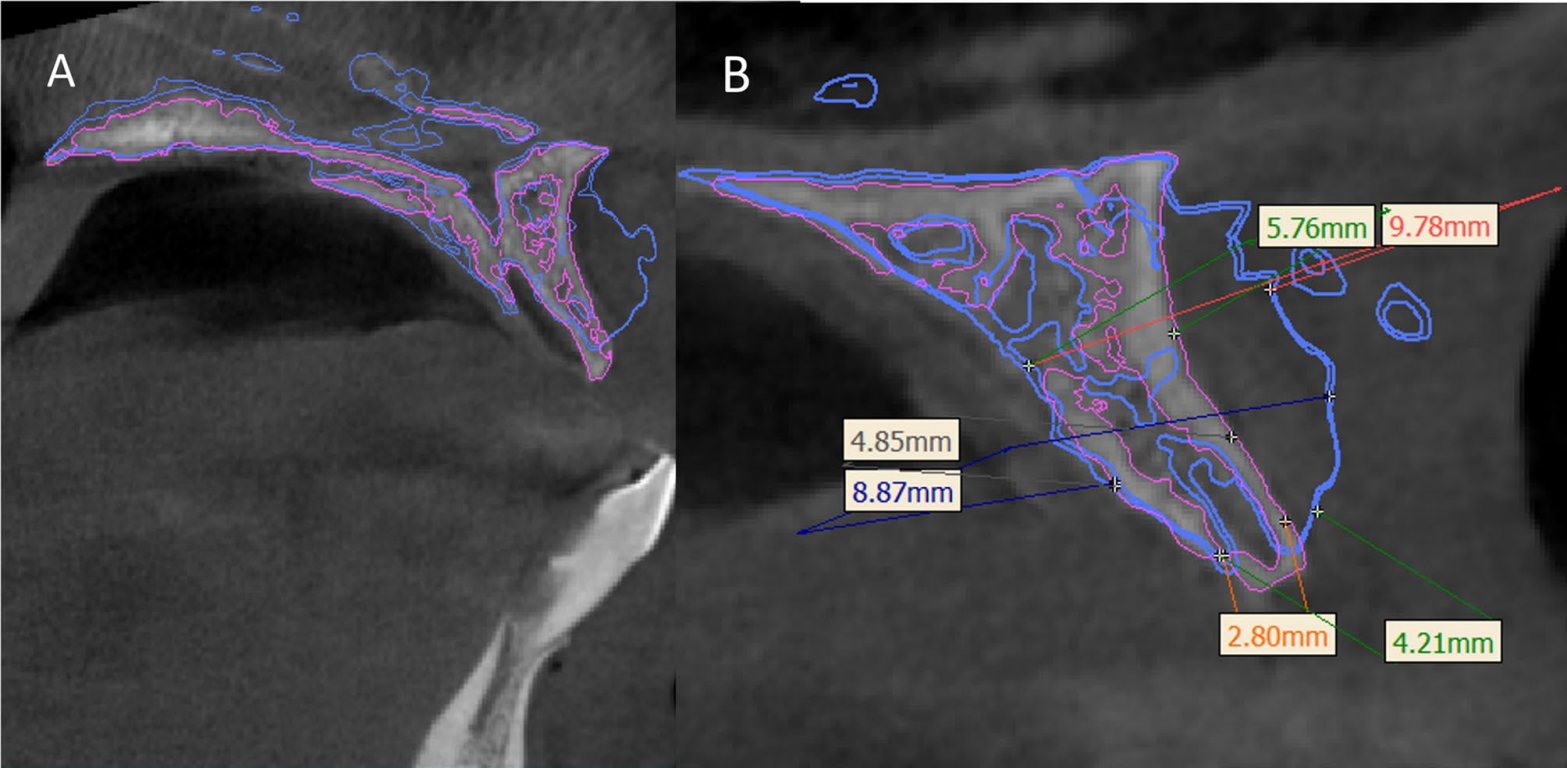
**A**) Standardization of assessment method for calculation of bone width by superimposition of preoperative CBCT (pink outline) and the 4 months post-operative CBCT (blue outline) **B**) bone width was measured at three different levels; at the crestal, at 5 mm and 10 mm

Using the “image registration” tool in mimics 21.0 (Materialise, Leuven, Belgium), superimposition was done to standardize the measurements between the preoperative and the four-month CBCT scans, where the anterior nasal spine, lateral nasal wall, and pterygoid plates were marked as fixed anatomical landmarks (Fig. 7). For each implant site, bone width was measured at three levels: the alveolar crest, at 5 mm and at 10 mm. Then the mean was calculated for the preoperative and the four-month postoperative periods. The mean gain in bone width was finally calculated.

The accuracy of the guided procedure in the study group was assessed by using the preoperative CBCT where the preoperative planning was superimposed over the immediate postoperative CBCT scan. Bone width was measured in both of them for each implant site in the same manner at three levels, then the average was obtained, and the difference was calculated.

*Radiographic assessment of bone width stability around the implants* took place by comparing the CBCT scans done at the time of implants exposure (four months after implant placement) with that done immediately following implant placement.

The final prosthetic loading was done using FP1 fixed zirconia restorations for all patients in both groups, and a *clinical assessment* of the gingival and biological integrity was done one year after delivery of the final prosthesis by recording the pink esthetic score (PES) for each implant in both groups, and calculating the mean for each case.

Mental nerve recovery assessment (recovery from the surgical adverse effects) was done as recommended by Tonetti et al. in the bone augmentation core outcome set and measurements *(BA-COSM)* [24]. It started three weeks following the first intervention to establish a baseline record, then repeated after two months. The medical research council (MRC) scale [25] was used as a reference. Pain, touch, and two-point discrimination were tested. The patients were seated comfortably and asked to keep their eyes closed, then a blunt-tipped caliper was set at 10 mm distance to start the two-point discrimination test. The distance was reduced gradually in 2 mm increments while the patients mentioned if one or two points were felt each time. A dental probe was used for the nociceptive stimulation test, starting with the upper lip as a reference. The patients were asked if they felt any pain. A cotton-tipped applicator was used for the light touch and directional stroke tests, starting also with the upper lip as a reference. It was repeated three times, and the patients were asked if they felt the cotton on their lower lip and about the direction of the stroke, respectively. Two out of three correct answers were regarded as a normal response. Moreover, subjective feedback was provided from the patients about the presence or absence of numbness, the surface area affected, and its resolution [26].

### Statistical methods

All data was collected, tabulated, and subjected to statistical analysis. Statistical analysis was performed by SPSS (Statistical Package for the Social Sciences- IBM Corp., Armonk, NY), while Microsoft Office Excel was used for data handling and graphical presentation. Quantitative variables (Horizontal Bone gain, Accuracy) were described by the mean and standard deviation (SD), while qualitative (categorical) variables (Gender) were described by frequencies and percentages. Data were explored for normality by checking the data distribution and using Kolmogorov-Smirnov and Shapiro-Wilk tests for further choice of appropriate parametric and non-parametric tests.

All the variables were found to be normally distributed, allowing the use of parametric tests. The independent sample t-test was applied to compare the means of the two groups. Qualitative data (gender) were compared using the chi-square test. The percent change was calculated by the formula: (value after-value before) / value before X100.

The significance level was considered at *P* < 0.05 (S). Two-tailed tests were assumed throughout the analysis for all statistical tests.

## Results

### Clinical results

A total of twenty patients (13 males and 7 females) were enrolled in this study; their ages ranged from 20 to 50 years old, and they were randomly allocated into two equal groups. In the study (computer-guided) group, the surgery was totally performed with the aid of bone supported cutting and positioning guides, while in the control group, all the steps were done in a freehand manner. The routine postoperative course of surgical edema resolved by the end of the first week in both groups.

In the study group, nine patients showed uneventful wound healing; however, the shadow of the fixation screws was observed in one patient after 2 months. It was closely followed up until the second stage, where the screws were removed and implants were placed normally. In the free-hand group, eight patients showed uneventful wound healing except for two cases that showed dehiscence, one of them after two weeks and the other after two months. The first one was managed by broad-spectrum antibiotics with irrigation and strict oral hygiene instructions, in addition to reopening the flap for periosteal scoring and tension-free closure after PRF application (platelet-rich fibrin). The second one was managed by graft reduction without raising a flap, strict oral hygiene instructions, and irrigation, with close follow-ups scheduled twice weekly. Both cases healed normally within a week.

Assessment of the *mental nerve recovery* (recovery from the surgical adverse effects) was done following the MRC scale (Table 1) as recommended by Tonetti et al. in their core outcome set and measurements *(BA-COSM)* for bone augmentation [24]using the two-point discrimination, touch, and pain tests. At three weeks postoperatively, all patients showed mild affection and scored S3 + in both groups. However, after two months, the second assessment was done and showed complete recovery in all patients in both groups (S4).

**Table 1:** MRC scale for neurosensory assessment

Grade	Description
S0	No sensation
S1	Deep cutaneous pain in an autonomous zone
S2	Some superficial pain and touch sensation
S2+	Superficial pain and touch sensation plus hyperesthesia
S3	Superficial pain and touch sensation without hyperesthesia; static two-point discrimination > 15 mm
S3+	Same as S3 with good stimulus localization and static two-point discrimination of 7–15 mm
S4	Same and S3 and two-point discrimination of 2–6 mm

MRC, Medical Research Council. Grades S3, S3+, and S4 indicate useful sensory recovery. Data from Birch R, Bonney G, Wynn-Parry CB. Surgical disorders of the peripheral nerves. Philadelphia: Churchill Livingstone, 1998: 405–414

**Fig. 8:**
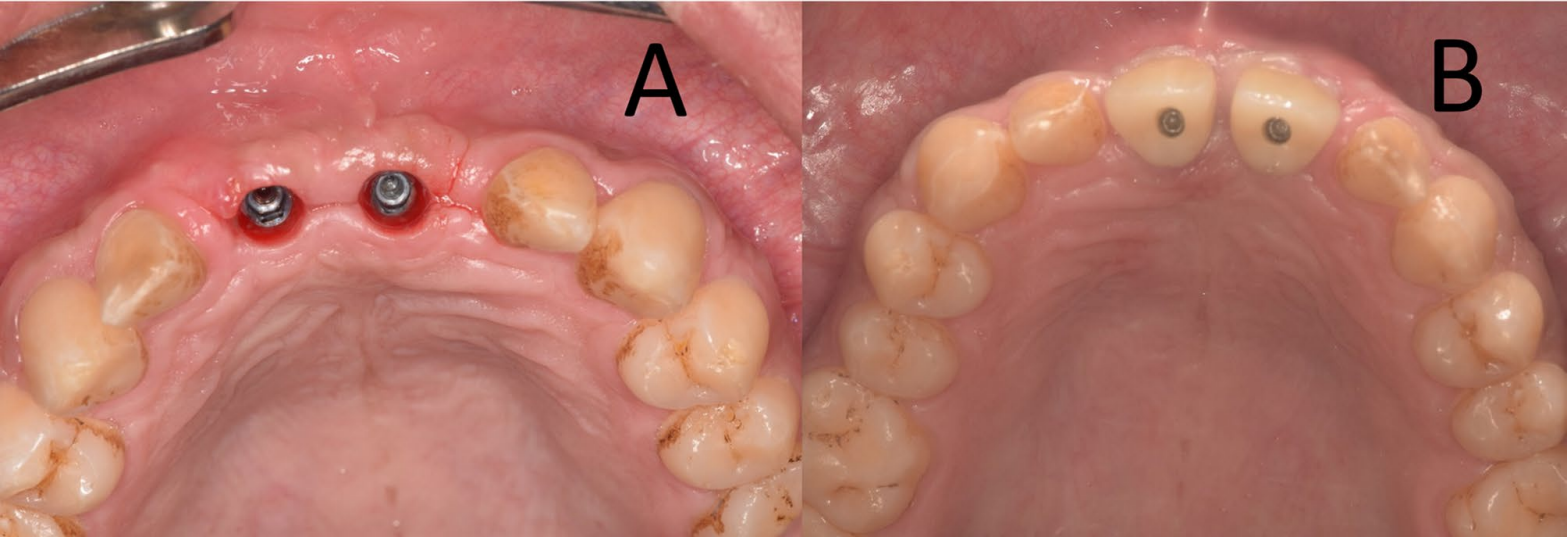
**A** and **B**, at the one-year recall visit

In both groups, a total of 45 endosteal dental implants (Dual Implants, Titan Industries, Cairo, Egypt) were placed successfully in the second stage surgery (four months after augmentation), leaving a sufficient thickness of bone, a minimum of 1–1.5 mm both buccally and palatally while at the same time being in a favorable position for the subsequent prosthetic loading. After four more months (eight months after the first intervention), all implants integrated successfully and the final prosthesis was delivered in all patients. Further recall visits were scheduled at six months and one year after delivery of the final prosthesis; clinical examination showed no abnormality, and all patients reported normal masticatory function and were satisfied with the cosmetic results. The mean PES, recorded at the one-year post-prosthetic loading recall visit as a *clinical assessment* of the gingival and biological integrity was 12.5 ± 1.03 for the study group and 11.7 ± 0.88 for the control group. (Fig. 8) (Table 2).

**Table 2:** Pink esthetic score values one year after prosthetic loading for all implants

Patient No.	Score per implant	Mean PES for each patient	
		STUDY group	CONTROL group
1 C	12		12.5
	13		
2 C	13		12.5
	12		
3 S	14	12.7	
	11		
	13		
4 S	13	13.5	
	14		
5 C	10		11
	12		
6 S	13	12.5	
	12		
7 S	10	10.7	
	12		
	10		
8 S	14	14	
	14		
9 C	10		10
	10		
10 C	11		11.3
	12		
	11		
11 C	10		11
	12		
12 C	12		11.5
	11		
13 C	10		12
	14		
14 S	13	13.5	
	14		
15 S	12	10.7	
	10		
	10		
16 C	12		12.5
	13		
17 S	12	12.5	
	13		
18 C	14		13
	13		
	12		
19 S	13	12.5	
	12		
20 S	12	12.5	
	13		
Mean / SD per group		12.5 ± 1.03	11.7 ± 0.88

### Radiographic result

The mean *horizontal bone gain* in the guided group was 3.66 ± 0.63 mm, which was higher than the Free hand group (3.33 ± 1.07 mm), yet the difference was not statistically significant (P-value = 0.4). (Table 3)

**Table 3:** Comparison of bone gain between both groups and the p-value of bone width changes around the implants at the time of exposure

Group	Minimum	Maximum	Mean	SD	Mean difference	*t* value	*p*-value	*p-value for bone width at the time of implant exposure*
Study	3.07	5.11	3.66	0.63	0.329	0.329	0.415 ns	0.656 ns
Control	0.78	4.57	3.33	1.07				

Significant level *p* ≤ 0.05, ns = non-significant

**Fig. 9:**
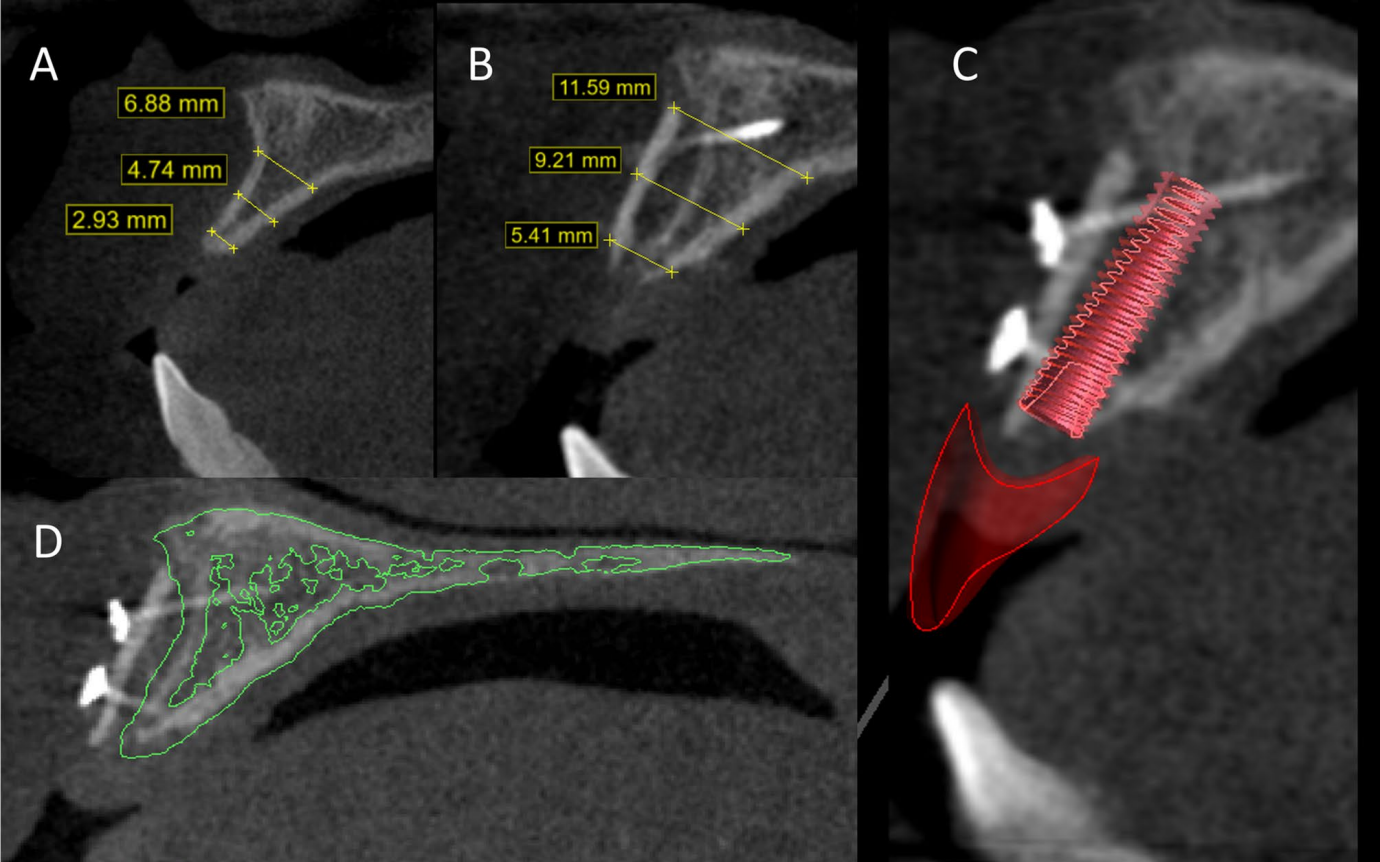
For the control group **A**) Preoperative view. **B**) ridge width after 4 months. **C**) implant planning with virtual prosthesis. **D**) superimposition between the preoperative CBCT (green outline) and the CBCT after 4 months

**Fig. 10:**
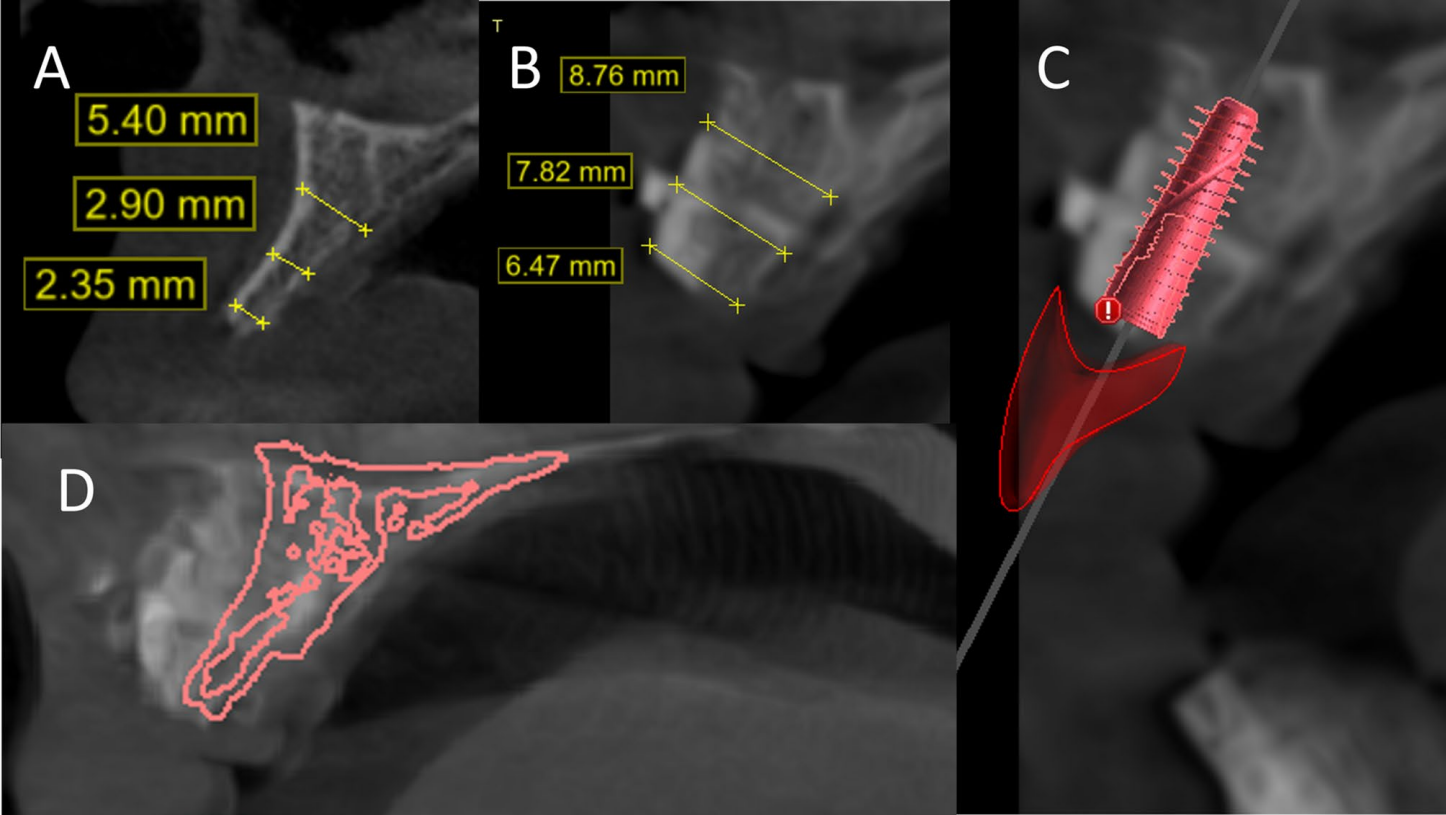
For the study group **A**) Preoperative view. **B**) ridge width after 4 months. **C**) implant with virtual prosthesis. **D**) superimposition between the preoperative CBCT (Pink outline) and the CBCT after 4 months

The mean *bone gain percentage* in the guided group was 105.71% ± 29.75%, which was higher than the mean value in the free hand group (90.41% ±31.77%), yet the difference was not statistically significant (P-value = 0.28). (Figures 9 and 10) (Table 4).

**Table 4:** Mean and standard deviation (SD) values for patient’s percentage of horizontal bone gain in both groups

Group	Minimum	Maximum	Mean	SD	Mean difference	*t value*	*p*-value
Study	73.86%	172.85%	105.71%	29.75%	0.153	1.112	0.281 ns
Control	17.79%	135.28%	90.41%	31.77%			

Significant level *p* ≤ 0.05, ns = non-significant

**Fig. 11:**
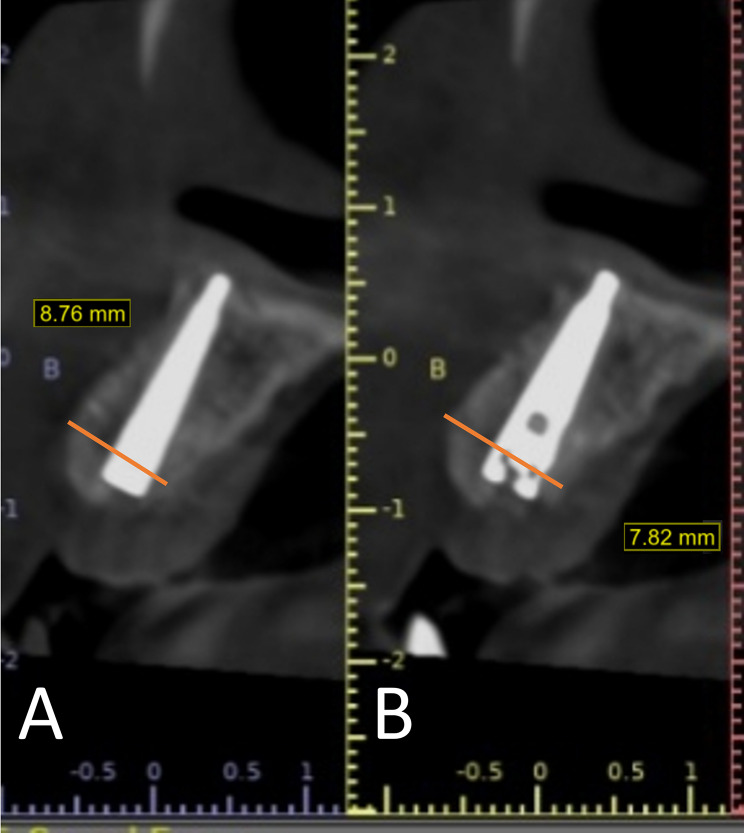
Horizontal bone width at the time of implant exposure for both groups (**A**: control and **B**: study)

**Fig. 12:**
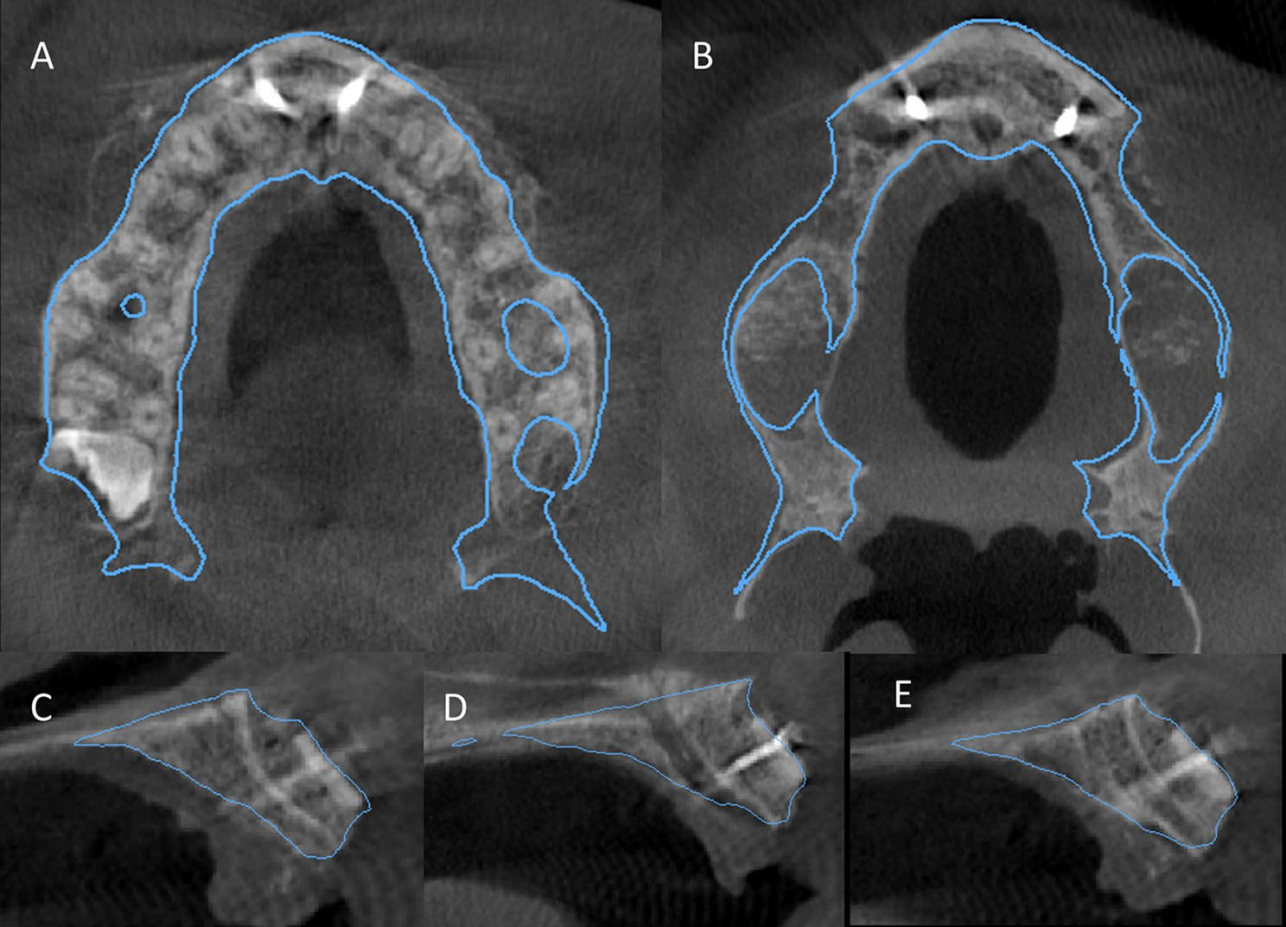
showing the assessment of accuracy in two different cases in the study group by superimposition between the preoperative plan (blue outline) and immediate post-operative CBCT. **A**, **B**) are axial views and **C**, **D**, **E** are different cross sections cuts

The stability of the augmented alveolar bone was measured four months after implant placement - at the time if implants exposure - for both groups to measure if any horizontal bone changes occurred around the implants *(radiographic stability)*, the difference between them was not statistically significant (P-value = 0.656) (Fig. 11), (Table 3). Regarding *the accuracy* in the study group, the mean value of the planned horizontal bone gain of the guided group (8.458 mm ± 0.567) was comparable to the mean value of the actual immediate postoperative bone gain (8.449 mm ± 0.566), and the difference was not statistically significant (P-value = 0.97). (Fig. 12) (Table 5).

**Table 5:** Assessment of accuracy; the virtual plan versus the immediate postoperative CBCT

Study Group	Mean	SD	Mean difference	*t value*	*p*-value
Planned Bone Gain	8.458	0.567	0.009	0.037	0.971 ns
Actual Immediate Postoperative Bone Gain	8.449	0.566			

Significant level *p* ≤ 0.05, ns = non-significant

## Discussion

Bone augmentation surgeries are technique sensitive, requiring a steep learning curve and a cumulative experience from the surgeon, especially the cortical shell technique, which requires not only harvesting of autogenous bone but its proper fixation at an adequate distance from the [1, 27–30] deficient ridge. Hence the hypothesis of the present study was that a fully computer-guided approach to harvest the bone shell and then to position and fix it would increase the predictability of the technique by increasing its accuracy.

In this study, the shells were harvested from the chin rather than the retromolar area because the unique contour and configuration of the anterior maxilla do not match the shape of straight retromolar shells, which may lead to sharp angles and difficulty in following the anatomical contour of the maxilla [16]. In another study [31]a total of thirteen mandibular blocks were harvested using virtually designed harvest guides and the results confirmed a safe operation regarding the nearby anatomical structures and allowed an increase in the dimensions of the graft when needed.

Also, a study by Osman et al. [7] reported ten cases of computer-guided symphyseal bone harvesting using patient-specific guides to minimize the risk of harm to adjacent structures, especially in cases of long anterior loops of the mental nerve or nearby root apices. However, still a disadvantage of any bony-supported guide is the need for a relatively larger exposure to ensure that the guide is accurately and passively seated, making use of the available surface anatomy or bony elevations and depressions. This wider exposure will produce tension on the mental nerves during retraction. For this very reason, we performed a neurosensory evaluation of the mental nerves in both groups. According to the MRC scale [25]all patients scored S3 + at the first assessment three weeks after the first operation, while the full recovery was documented two months later (S4). This mild affection could be attributed to the unavoidable tension due to retraction in both groups.

Cases of altered sensation in the mandibular anterior teeth were reported by many authors to show improvement over time and hence they are not permanent [10, 32]. Despite that, in the mentioned study by Osman et al. [7] the authors performed pulp testing to the mandibular anterior teeth to ensure the safety of the guided chin harvest on the pulp vitality of the adjacent teeth, all the pulp sensitivity tests in their guided group were normal. That’s why in the present study we didn’t perform pulp sensitivity tests and were satisfied by the reported safety of the harvest guide and followed the same safe distances used by them while designing the harvest guide; to locate the osteotomies at least 5 mm away from the apices of the anterior teeth, the inferior border and the mental foramen.

In the present study, a 1:1 particulate mixture of autogenous bone and xenograft was used to obtain better outcomes according to the findings of Mauro Merli et al. [33] who conducted a comparative study between using autologous bone alone as a gold standard and using a 50–50 mixture of xenogeneic and autogenous bone. The reported benefits of the 1:1 ratio included the presence of viable osteogenic cells while decreasing the amount of harvested bone, less discomfort, and a lower graft resorption rate.

The clinical results showed flap dehiscence in two cases in the control group (28.5%) which is a common complication after alveolar ridge augmentation as reported. this finding was similar to the finding of Horia Barbu et al. [34] in their study of horizontal augmentation using sticky bone versus bone shell technique, and the outcome indicate that ten cases out of the eighty participants included in their study showed post-operative flap dehiscence which caused graft failure. This finding was explained to be due to the use of large shell size and fixation of the cortical shell at a large distance from the native bone for over-correction, even when appropriate flap advancement and passive closure have been performed.

The difference in the mean bone gain between the study group (3.8 ± 0.7 mm) and the control group (3.0 ± 1.3 mm) was not statistically significant. However, it could be justified by better and more precise positioning of the graft in the computer-guided group, which had a better impact on the final ridge contour. This conclusion is similar to Ning Zhu et al. [35] who compared the effectiveness of a fully digitalized technique in controlling the contour of onlay grafts versus the conventional protocol. The digital workflow was found to be more precise, efficient, and predictable.

Additionally, other authors conducted a comparative study between computer-guided lateral ridge augmentation versus freehand GBR [36]. The bone thickness was measured crestally and at 5 mm and the result showed a significantly greater bone thickness in the study group than in the control group with the median labial thickness amount of 4.31 mm (mean 4.3 ± 0.7 mm) in the test group and 2.9 mm (mean 2.9 ± 1.02 mm) in the control group. In another study, the same authors also introduced the application of customized surgical templates to achieve a proper graft contour in guided bone regeneration [37].

In the current study, accuracy of the computer-guided approach was assessed by superimposition between the preoperative plan and the immediate post-operative CBCT scan. The results showed that the mean planned horizontal bone gain in the guided group (8.458 ± 0.567 mm) was comparable to the mean value of the actual immediate postoperative bone gain (8.449 ± 0.566 mm), and the difference was not statistically significant (P-value = 0.97), concluding that the guide was reliable until the final fixation of the shell and prevented any noticeable difference from the planned position. The results were also comparable to that reported with splint-less Le Fort 1 osteotomy in orthognathic surgery [38]and with the reconstruction of mandibular contour defect using patient-specific titanium mesh [39].

In the present study, the surgical stents were used for less than 60 min and hence they could be classified as “class I”: “transient” devices. Since the material of the guides is heat sensitive, the CDC approved some chemical sterilants [40] such as glutaraldehyde ≥ 2% with contact time of 10 h. In our study we kept both the harvest and the positioning guides in contact with glutaraldehyde 2% for 12 h (overnight).

## Conclusion

From the results of the present study, there was no statistically significant difference between both groups with regard to horizontal bone gain. However, it can be concluded that computer-guided harvest and fixation of autogenous cortical shells could be a precise and safe alternative for the conventional free hand protocol that is worth further investigations to explore its different applications.

## Electronic supplementary material

Below is the link to the electronic supplementary material.

Supplementary Material 1

## Data Availability

The data that support the findings of this study are available from the corresponding author upon reasonable request.
